# Cost-Effectiveness of Supervised versus Unsupervised Rehabilitation for Rotator-Cuff Repair: Systematic Review and Meta-Analysis

**DOI:** 10.3390/ijerph17082852

**Published:** 2020-04-21

**Authors:** Umile Giuseppe Longo, Alessandra Berton, Laura Risi Ambrogioni, Daniela Lo Presti, Arianna Carnevale, Vincenzo Candela, Giovanna Stelitano, Emiliano Schena, Ara Nazarian, Vincenzo Denaro

**Affiliations:** 1 Department of Orthopedic and Trauma Surgery, Campus Bio-Medico University, Via Alvaro del Portillo, 200, Trigoria, 00128 Rome, Italy; a.berton@unicampus.it (A.B.); laura.ambrogioni@gmail.com (L.R.A.); d.lopresti@unicampus.it (D.L.P.); v.candela@unicampus.it (V.C.); g.stelitano@unicampus.it (G.S.);; 2 Research Unit of Measurements and Biomedical Instrumentation, Campus Bio-Medico University, Via Alvaro del Portillo, 200, Trigoria, 00128 Rome, Italy; arianna.carnevale@unicampus.it (A.C.); e.schena@unicampus.it (E.S.); 3 Center for Advanced Orthopedic Studies, Carl J. Shapiro Department of Orthopedic Surgery, Beth Israel Deaconess Medical Center, Harvard Medical School, Boston, MA 02215, USA; anazaria@bidmc.harvard.edu; 4 Department of Orthopedic Surgery, Yerevan State Medical University, Yerevan 0025, Armenia

**Keywords:** supervised, unsupervised, exercise, cost analysis, rehabilitation, rotator cuff

## Abstract

Background: The objective of the present study was to compare the efficacy between supervised and unsupervised rehabilitation after rotator-cuff (RC) repair in terms of clinical outcomes, visual-analog-scale (VAS) score, range of motion (ROM), and risk of retear. Material: a comprehensive search of Pubmed, CINAHL, Cochrane, EMBASE, Ovid, and Google Scholar databases through a combination of the following keywords with logical Boolean operators: “informed”, “uninformed”, “unsupervised”, “supervised”, “rehabilitation”, “physical therapy”, “physical therapies”, “postoperative period”, “physical-therapy techniques”, “physical-therapy technique”, “exercise”, “exercise therapy”, “rotator cuff”, “rotator-cuff tear”, and “rotator-cuff repair”. For each article included in the study, the following data were extracted: authors, year, study design, sample size and demographic features, RC tear characteristics, clinical outcomes, ROM, VAS score, retear rate, and time of follow-up. Meta-analysis was performed in terms of VAS score. Results: Four randomized control trials with 132 patients were included. One study demonstrated significant improvement in VAS, active ROM, and the activity of the muscle’s motor units at stop and during maximal effort in supervised patients. Another one showed lower retear rates in the supervised group. The remaining two randomized controlled trials did not reveal any significant differences between supervised and unsupervised rehabilitation in terms of clinical outcomes. Moreover, higher costs were described for supervised rehabilitation. The VAS was not significantly different in the two groups (9.9 compared with 8.25, p = 0.23). Conclusions: although several publications address the problem of RC lacerations, there is a paucity of evidence in the literature regarding the effectiveness of supervised and unsupervised rehabilitation protocols. This systematic review and meta-analysis showed no significant differences between the two types of rehabilitation in terms of VAS scores, while outlining the pros and cons of each protocol.

## 1. Introduction

Rotator-cuff (RC) tear is a common shoulder disease, representing the leading cause of orthopedic evaluations in the U.S. population [1].

RC tear treatment increasingly demands a surgical approach, causing increases in healthcare expenditures related to its management [2,3,4]. In particular, the most recent national registry study showed that approximately 65% of all annual RC repairs are performed in patients <65 years of age [5,6]. This condition with increasing incidence in the active population explain the critical socioeconomic burden that should be considered [3,4]. Several studies are currently ongoing to evaluate the potential superiority of conservative treatment over a surgical approach [7,8,9,10,11,12]. Although much of the healthcare expenditure is due to intervention and hospitalization, the economic burden of the rehabilitation period may be underestimated [1].

Surgery alone is often inadequate to guarantee the satisfactory return of complete shoulder function. In the postoperative period, patients often complain of limitations in active movements, and pain in the neck and the involved muscles [13]. Therefore, a correct rehabilitation period, aimed to restore the shoulder function and avoid the risk of retear, is essential [14]. An efficient rehabilitation protocol may reduce the economic implications of the postoperative treatment of RC surgery [3,15,16]. Indeed, several studies were designed to realize a program that is as short as possible, allowing patients to return to their daily activities as soon as possible [17,18,19,20,21].

However, little information is available concerning the superiority of supervised rehabilitation, which requires multiple physiotherapy sessions, compared to unsupervised rehabilitation at home [1,15,22,23,24,25]. In both cases, there could be advantages in the management of this disease. If the evidence were in favor of supervised rehabilitation, more could be invested in the rehabilitation period to reduce the risk of further interventions. On the other hand, the absence of differences between the two forms of rehabilitation could reduce costs associated with physiotherapy.

Therefore, the first objective of the present study was to compare the efficacy of supervised and unsupervised rehabilitation protocols post-RC repair in terms of clinical outcomes, visual-analog-scale (VAS) score, range of motion (ROM), and risk of retear. The second objective was to conduct cost–benefit analysis between receiving rehabilitation sessions supervised by physiotherapy or self-care rehabilitation.

## 2. Material and Methods

### 2.1. Literature-Search Strategy and Study Selection

To achieve the objectives of this systematic review and meta-analysis, a comprehensive search of the Pubmed, CINAHL, Cochrane, EMBASE, Ovid, and Google Scholar databases was performed according to the Preferred Reporting Items for Systematic Reviews and Meta-Analyses (PRISMA) guidelines with a PRISMA checklist and algorithm [26,27]. The combination of the following keywords with logical Boolean operators has been used to fulfil this in-depth research: “informed”, “uninformed”, “unsupervised”, “supervised”, “rehabilitation”, “physical therapy”, “physical therapies”, “postoperative period”, “physical-therapy techniques”, “physical-therapy technique”, “exercise”, “exercise therapy”, “rotator cuff”, “rotator-cuff tear”, and “rotator-cuff repair”. The search strategy was (informed OR uninformed OR unsupervised OR supervised OR rehabilitation OR “physical therapy” OR “physical therapies” OR “postoperative period” OR “physical-therapy techniques” OR “physical-therapy technique” OR exercise OR exercises OR “exercise therapy” OR “exercise therapies”) AND (“rotator cuff” OR “rotator-cuff tear” OR “rotator-cuff repair”) AND “rotator-cuff repair”). Following the inclusion criteria, the search was filtered by clinical trial, and all retrieved results from the inception of these databases until March 2020 were included. Three independent reviewers (U.G.L., L.R.A., and V.D.) ascertained the eligibility of these studies on the basis of the significance of the title and abstract without excluding any journal. Studies with missing abstracts or that did not contain the necessary information to define their eligibility were examined in their entirety. Due to the language skills of the reviewers, all studies in English, French, Dutch, Turkish, Spanish, and Italian were potentially suitable for the study. A cross-reference of the studies included in this systematic review and meta-analysis was further carried out to search for other suitable articles for this study. The same three reviewers collected data independently to minimize the risk of bias. Where reviewers differed on eligibility criteria or data retrieval, the senior investigator (V.D.) made the final decision.

To be included in this mixed-method review, eligible studies had to meet the following inclusion criteria: (i) the study design had to be a randomized controlled trial, (ii) the study had to compare the supervised and unsupervised rehabilitation groups, (iii) the study population had to undergo RC repair, and (iv) the studies had to be published in a peer-reviewed magazine or presented in a conference. Exclusion criteria were: (i) the study design being different from a randomized controlled trial (i.e., reviews, case reports, articles on animals, cadavers, or in vitro researches, biomechanical reports, technical notes, letters, and instructional studies), (ii) randomized controlled trials that addressed a single type of intervention (either self-care rehabilitation or exercises supervised by the physiotherapist), and (iii) the study population included patients with other RC diseases (e.g., RC tendinopathies and shoulder-impingement syndrome). To cope with the lack of studies available in the literature, it was not possible to differentiate by lesion size, traumatic or nontraumatic event, acute or chronic and partial or complete tear. Furthermore, for the same reason, the type of surgical procedure (i.e., open, miniopen, or arthroscopy) was not considered as a criterion for inclusion.

### 2.2. Data-Extraction Process

The following data were extracted from each article included in the study: authors, year, study design, sample size and demographic features, RC tear characteristics, clinical outcomes, ROM, VAS score, retear rate, and time of follow-up.

Patient-Reported Outcome Measures (PROMs), the Constant–Murley score (CMS), the American Shoulder and Elbow Surgeons (ASES) score, the Disabilities of the Arm, Shoulder, and Hand (DASH) score, the University of Pennsylvania (UPenn) score, the Shoulder Pain and Disability Index (SPADI) score, and the University of California Los Angeles (UCLA) shoulder score have been widely adopted to observe the response to surgical RC repair. The Beck depression inventory (BDI) measures the intensity of the state of depression and anxiety, especially in moderately depressed patients. It therefore allows to monitor overall benefits provided by surgical intervention. The VAS pain score is used to quantify the severity of pain perception in patients. The scale ranges from 0 (“no discomfort”) to 10 (“maximal discomfort”).

### 2.3. Meta-Analysis

A comparison between the groups was performed with different clinical outcomes among the randomized controlled trials. Therefore, meta-analysis was only possible in terms of VAS score at rest, which was the only common score for all studies. Review Manager (RevMan, version 5 for Windows; Cochrane Information Management System) software was used to evaluate the magnitude of therapy outcome. We measured an I^2^ index as an assessment of heterogeneity for primary analysis. The I^2^ value describes the percentage of the entire change between articles, which is made by heterogeneity rather than by chance. We estimated the low I^2^ index as ≤25% and the high I^2^ index as ≥75%. Categorical variable data were described as rate with percentage. Continuous variable data were included as mean index with a range across minimal and maximal values; *p* < 0.5 was always deemed statistically meaningful.

### 2.4. Quality Assessment

To evaluate the pertinence of the chosen articles, and to assess the power of recommendation of the treatment that was offered in the published studies, Grading of Recommendations Assessment, Development, and Evaluation (GRADE) was employed. GRADE is utilized to rate the body of evidence at the outcome level rather than the study level. Quality is assessed on four elements: study design, study quality, consistency, and directness. The mixture of these factors defines the power of recommendation provided with the qualitative evaluation of the evidence: high, moderate, low, and very low quality. For each of risk of bias, imprecision, inconsistency, indirectness, and publication bias, authors must make a judgement about whether the risk of bias in individual studies is sufficiently large that their confidence in the estimated treatment effect is lower. Therefore, authors have the option of decreasing their level of certainty one or two levels (e.g., from high to moderate) [28].

## 3. Results

**Figure 1:**
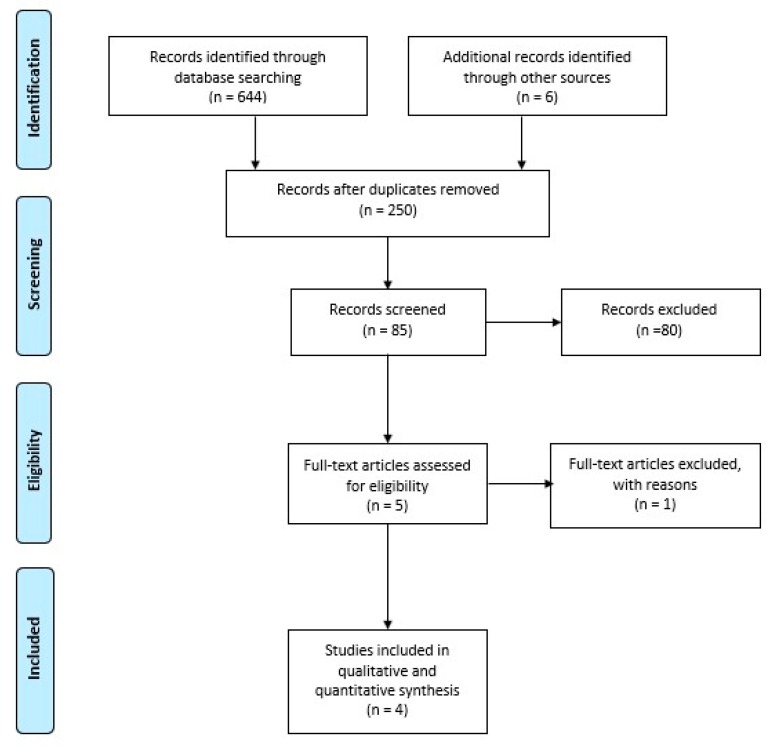
Preferred Reporting Items for Systematic Reviews and Meta-Analyses (PRISMA) 2009.

The research strategy adopted for the study yielded 650 results. After the removal of duplicates, 250 articles were filtered by study design (i.e., clinical studies). Research and cross-references resulted in a total of 85 references, 81 of which were discarded due to the absence of relevance to the study topic, not meeting the inclusion criteria, or both (Figure 1). At the end of the study-selection process, four randomized controlled trials were selected for meta-analysis [22,23,24,25]. Supervised rehabilitation was defined as physiotherapy consisting of multiple sessions with a physical therapist, whereas unsupervised rehabilitation as home exercises is performed without the supervision of a physical therapist. For this reason, a potentially suitable study was excluded given that the group considered unsupervised home exercises as those where the patients viewed a videotape by the physical therapist who, in turn, monitored their progress [15]. Therefore, four randomized controlled trials were included in this qualitative and quantitative synthesis (Figure 1).

### 3.1. Demographics

The overall sample size included 132 patients who underwent surgical RC repair. Subjects were categorized into two groups, supervised (64 patients) and unsupervised (68 patients). The average age in the first group was 56.9 years, ranging from 15 [25] to 81 [24] years, whereas the average age in the latter was 58.9 years, ranging from 19 [25] to 83 [24]. Regardless of study group, the average time of follow-up was 13 weeks, ranging from 5 [25] to 24 weeks [15,24]. Study characteristics are detailed in Table 1.

### 3.2. RC Tear Characteristics

The size of the lesions was extracted in three out of the four studies, for a total of 104 patients (Table 1) [15,22,24,25]. The mean size of the lesions was 2.56 cm: 2.43 cm in the supervised group [22,24,25] and 2.69 cm in the unsupervised group [22,24,25]. The number of torn RC tendons was reported in 1 of 4 studies, for a total of 58 patients (Table 1) [15,24]. The rupture of one tendon was observed in 32% of subjects in the supervised and 34% in the unsupervised group; the rupture of two tendons was observed in 10% and 12%, respectively; the rupture of three tendons in 4% and 8%, respectively; the rupture of four tendons was not observed.

### 3.3. Clinical Outcomes

Several clinical outcomes were described in the chosen studies. The Constant–Murley (CMS), American Shoulder and Elbow Surgeons (ASES), Disabilities of the Arm, Shoulder, and Hand (DASH), University of California at Los Angeles (UCLA), Beck depression inventory (BDI), Shoulder Pain and Disability Index (SPADI), and University of Pennsylvania (UPenn) scores were reported in different randomized controlled trials, with no possibility to compare them. The clinical scores used in the studies are detailed in Table 2.

### 3.4. Range of Motion

ROM was analyzed in terms of forward flexion, abduction, and external rotation in one of the four articles (Table 2) [24].

### 3.5. VAS Score

Pain was analyzed in three of the four randomized controlled trials (Table 2) [22,23,25]. VAS score was analyzed in 74 shoulders: mean value of the supervised group (38 subjects) and the unsupervised group (36 patients) was 9.9 and 8.25, respectively.

### 3.6. Meta-Analysis

**Figure 2:**
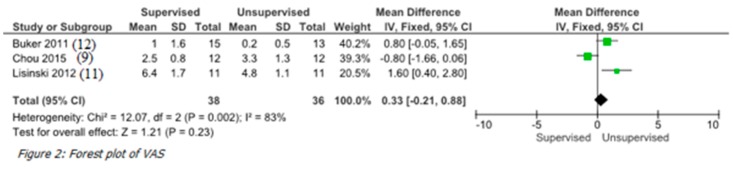
Forest plot of VAS score.

Quantitative synthesis was performed in terms of VAS score at rest in three out of the four studies [22,23,25]. We discovered no important differences between the groups in terms of pain control (*p* = 0.23; Figure 2).

### 3.7. Quality Assessment

**Figure 3:**
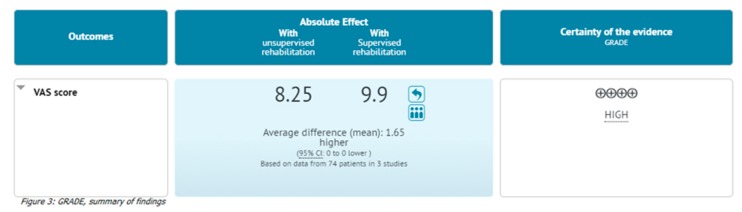
GRADE, summary of findings.

The appropriateness of the chosen articles was discovered to be relevant (Figure 3). The decisions were reinforced by the whole exploration and comprehensive clinical-eligibility criteria. In the GRADE system of rating quality of evidence for every result, randomized trials start as high-quality evidence, even if they could be considered low by one or more of the five classes of limits.

Interests regarding publication mistakes were derived from the paucity of clinical trials, and the variable recording of results between trials increasds the chance of a particular reporting bias. However, we did not evaluate signs of publication bias or discriminating recording bias. The power of inference was restricted.

## 4. Discussion

RC repair is a common orthopedic procedure that always requires an adequate postoperative rehabilitation protocol [14]. Failures of RC repair arise when tendon retear or joint stiffness occurs [2,29,30]. Recent studies showed that early rehabilitation is more likely to cause a rerupture of the tendon, whereas less intensive rehabilitation may cause shoulder stiffness [31,32]. Therefore, the best physical-rehabilitation protocol should be designed in order to balance the risk of retear with the risk of stiffness, and thus provide the highest quality of care at the lowest possible cost [1,14,30]. However, there is not enough evidence supporting the possible superiority between supervised rehabilitation or individual physical sessions in terms of clinical outcomes [1]. Evidence in favor of the former or latter form of rehabilitation could benefit the patient and reduce healthcare costs [1].

The present study has a high level of evidence, since only randomized controlled trials comparing supervised and unsupervised rehabilitation after RC repair were recruited. On the other hand, up to now, only four studies are available in the literature, highlighting how little is known about postoperative rehabilitation, even if its role in intervention success is incontrovertible. Furthermore, the available studies reported heterogeneity in terms of follow-up, outcome measures (clinical outcomes, re-extraction rate, neurophysiological examination), and physical exercises, suggesting that there is currently no consensus for the rigorous evaluation of the rehabilitation protocol. Therefore, definitive conclusions about the best form of physical therapy could not be reached.

The previous systematic review by Dickinson et al. regarding the cost effectiveness of postoperative rehabilitation for patients undergoing RC repair included the study by Roddey et al. [1,15]. Although this study compared two forms of rehabilitation, it was not included in our quantitative synthesis because the group performing exercises at home received instructions through videotapes. Therefore, it cannot be considered a truly unsupervised rehabilitation protocol according to our definition. The efficacy between supervised and unsupervised rehabilitation after RC repair in terms of clinical outcomes, VAS score, ROM, and risk of retear was evaluated in all the selected studies. However, due to the heterogeneity of scores used in the four randomized controlled trials, meta-analysis could only be performed in terms of VAS scores. Even though Lisinski et al. showed significant improvement of pain level (VAS), active ROM, activity of muscle motor units at stop and throughout maximal exercise only in supervised patients, the present meta-analysis demonstrated no significant differences between supervised and unsupervised rehabilitation [25]. Furthermore, two of the four studies included in this quantitative synthesis showed no difference between the groups, raising the question of whether the cost of supervised rehabilitation is justified [23,24].

Given the growing interest in personalized medicine, the role of the physical therapist in rehabilitation is strongly advocated by those who support the need for continuous monitoring of the patient’s postoperative progress [14,21,33,34]. Indeed, not only type of injury, but also several parameters, such as tear size and location [35], surgical technique [36,37,38,39], and concomitant pathologies [21,30,40], may influence the recovery of shoulder function [24,41]. Therefore, only an individualized and supervised rehabilitation program can consider all patient- and surgical-based variables on the basis of which specific exercise programs could be designed [13,39]. However, Hayes et al. showed comparable outcomes in patients who had undergone individualized, supervised rehabilitation, and patients who had performed standard unsupervised exercises at home [24]. This suggests that supervision alone is not enough to achieve significant improvements, even though the presence of a physical therapist allows the immediate identification and correction of rehabilitation mistakes, potentially leading to better results [4,34]. Indeed, Chou et al., combining supervised rehabilitation with a monitoring device during the first few weeks, showed a lower retear rate in patients treated by a standardized rehabilitation protocol performed at home [22]. Unfortunately, if supervised rehabilitation is already a cost, further devices that improve clinical outcomes are an additional expense.

On the other hand, evidence showed that unsupervised rehabilitation improves clinical results without introducing an additional burden on healthcare costs. Buker et al. analyzed the cost effectiveness of 15 patients treated with supervised physical therapy and 13 patients treated with a home-based exercise program [23]. Costs related to treatment procedures were reported, such as those for TENS application, ultrasound application, joint-motion exercises, progressive resistant exercises, and total session fees [42]. They found no statistical variations between the two groups in terms of pain, functional state, quality of life, and depression status, but the supervised-physical-therapy group reported higher costs. Thus, it should make us wonder whether the slight benefit to the patient from supervised rehabilitation is enough to cover its cost.

However, the two studies that reported the superiority of supervised rehabilitation are those that conducted more in-depth analysis with neurophysiological examination or with the inclusion of devices in the first postoperative weeks [22,25]. These findings open the possibility that the evaluation of effectiveness between the two forms of rehabilitation cannot only be based on the application of different, and therefore incomparable, clinical scores among studies [12]. Thus, these results can be used for future randomized controlled trials to employ outcome measures that are comparable to those already available in the literature. In this way, future meta-analyses could consider not only the VAS pain score, but also ROM and PROMs, adding significant information in this relevant topic of orthopedics.

Interestingly, only one study reported a scale for evaluating depression, demonstrating the importance of the mental and human dimension of care, which tends to be underestimated. For instance, sleep disorders are among the most frequent complaints of patients with RC tears. Recent studies highlighted the impact that other human variables, such as sleep quality, patient expectation, cognitive, affective psychologic factors or distress, and socioeconomic status may have on the clinical results of RC tears [13,43,44,45,46,47,48,49,50,51].

## 5. Conclusions

The definition of an effective treatment of patients undergoing RC repair in terms of recovery costs can contribute to health-service planning. Unfortunately, although several publications address the problem of RC lacerations, there is a paucity of evidence in the literature on the effectiveness of supervised and unsupervised rehabilitation protocols. This systematic review and meta-analysis did not demonstrate significant differences between the two forms of rehabilitation in terms of VAS scores, and has highlighted the pros and cons of both forms of rehabilitation. Therefore, on the basis of the available evidence, no definitive conclusions can be drawn. A future perspective would be the remote monitoring of home rehabilitation with new technological tools. This would combine the advantages of supervised physiotherapy, intervening efficiently in postoperative rehabilitation, and the lower cost of unsupervised therapy. Further studies should also investigate the potential role of the psychological factor in RC tears to develop new therapeutic approaches.

## Figures and Tables

**Table 1:** Demographics and RC Tear characteristics.

Authors	Study Design	No. of Patients	Rehabilitation Form (Shoulder No.)	Sex		Mean Age ± SD (Range, Year)	Dominant (%)	Not Dominant (%)	Size of Lesion ± SD (Range, cm)	Time of Follow-Up (Weeks)	No. of Rotator-Cuff Tendons Torn			
				M (%)	F (%)						1	2	3	4
Buker N. et al., 2011 [23]	Randomized clinical trial (II)	28	Supervised (15)	5 (18%)	23 (82%)	59.8 ± 9.1 (40–83)	-	-	-	12	-	-	-	-
			Unsupervised (13)				-	-	-		-	-	-	-
Chou C. et al., 2015 [22]	Randomized clinical trial (II)	24	Supervised (12)	4 (33%)	8 (67%)	65.1 ± 8.7	-	-	2.42 ± 1.10	12	-	-	-	-
			Unsupervised (12)	3 (25%)	9 (75%)	67.9 ± 9.6	-	-	2.8 ± 1.47		-	-	-	-
Hayes K. et al., 2004 [24]	Randomized clinical trial (II)	58	Supervised (26)	20 (77%)	6 (23%)	58 ± 10 (41–81)	20 (77%)	6 (23%)	2.23 ± 2.64 (1–5.2)	24	18	6	2	0
			Unsupervised (32)	20 (63%)	12 (37%)	62 ± 11 (42–83)	19 (59%)	13 (41%)	2.45 ± 2.82 (1–5.47)		20	7	5	0
Lisinski P. et al., 2012 [25]	Randomized clinical trial (II)	22	Supervised (11)	6 (55%)	5 (45%)	45 (15–60)	-	-	2.66 (1–5)	5	-	-	-	-
			Unsupervised (11)	6 (55%)	5 (45%)	46 (19–50)	-	-	2.83 (1–5)		-	-	-	-

**Table 2:** Clinical outcomes, range of motion (ROM), and visual-analog-scale (VAS) score. Note: SPADI, Shoulder Pain and Disability Index; UPenn, University of Pennsylvania; ASES, American Shoulder and Elbow Surgeons; UCLA, University of California at Los Angeles; DASH, Disabilities of the Arm, Shoulder, and Hand; BDI, Beck depression inventory.

Authors	Rehabilitation Form (Shoulder No.)	Clinical Outcomes							ROM			VAS Score
		Constant–Murley Score	SPADI	UPenn	ASES	UCLA	DASH	BDI	Forward Elevation ± SD (Range, Degrees)	Abduction ± SD (Range, Degrees)	External Rotation ± SD (Range, Degrees)	
Buker N. et al., 2011 [23]	Supervised (15)	51.53 ± 10.69	-	-	-	-	-	7.8 ± 4.16	-	-	-	1.00
	Unsupervised (13)	72.23 ± 7.35	-	-	-	-	-	7.77 ± 6.69	-	-	-	0.15
Chou C. et al., 2015 [22]	Supervised (12)	22.6	-	-	73.2	24.2	42.4	-	-	-	-	2.5
	Unsupervised (12)	27.7	-	-	62.6	17.9	45.4	-	-	-	-	3.3
Hayes K. et al., 2004 [24]	Supervised (26)	-	-	-	-	-	-	-	150 (142–158)	142 (130–154)	51 (46–56)	-
	Unsupervised (32)	-	-	-	-	-	-	-	144 (132–156)	130 (117–143)	43 (36–50)	-
Lisinski P. et al., 2012 [25]	Supervised (11)	-	-	-	-	-	-	-	-	-	-	6.4
	Unsupervised (11)	-	-	-	-	-	-	-	-	-	-	4.8
